# Ototoxicity and Platinum Uptake Following Cyclic Administration of Platinum-Based Chemotherapeutic Agents

**DOI:** 10.1007/s10162-020-00759-y

**Published:** 2020-06-24

**Authors:** Benjamin K. Gersten, Tracy S. Fitzgerald, Katharine A. Fernandez, Lisa L. Cunningham

**Affiliations:** 1 Section on Sensory Cell Biology, National Institute on Deafness and Other Communication Disorders, National Institutes of Health, Bethesda, MD 20814 USA; 2 Mouse Auditory Testing Core, National Institute on Deafness and Other Communication Disorders, National Institutes of Health, Bethesda, MD 20814 USA; 3 Porter Neuroscience Research Center, 35A Convent Drive, Room 1D-955, Bethesda, MD 20892 USA

**Keywords:** cisplatin, carboplatin, oxaliplatin, ICP-MS

## Abstract

Cisplatin is a widely used anti-cancer drug used to treat a variety of cancer types. One of the side effects of this life-saving drug is irreversible ototoxicity, resulting in permanent hearing loss in many patients. In order to understand why cisplatin is particularly toxic to the inner ear, we compared the hearing loss and cochlear uptake of cisplatin to that of two related drugs, carboplatin and oxaliplatin. These three drugs are similar in that each contains a core platinum atom; however, carboplatin and oxaliplatin are considered less ototoxic than cisplatin. We delivered these three drugs to mice using a 6-week cyclic drug administration protocol. We performed the experiment twice, once using equimolar concentrations of the drugs and once using concentrations of the drugs more proportional to those used in the clinic. For both concentrations, we detected a significant hearing loss caused by cisplatin and no hearing loss caused by carboplatin or oxaliplatin. Cochlear uptake of each drug was measured using inductively coupled plasma mass spectrometry (ICP-MS) to detect platinum. Cochlear platinum levels were highest in mice treated with cisplatin followed by oxaliplatin, while carboplatin was largely excluded from the cochlea. Even when the drug doses were increased, cochlear platinum remained low in mice treated with oxaliplatin or carboplatin. We also examined drug clearance from the inner ear by measuring platinum levels at 1 h and 24 h after drug administration. Our findings suggest that the reduced cochlear platinum we observed with oxaliplatin and carboplatin were not due to increased clearance of these drugs relative to cisplatin. Taken together, our data indicate that the differential ototoxicity among cisplatin, carboplatin, and oxaliplatin is attributable to differences in cochlear uptake of these three drugs.

## Introduction

**Fig. 1:**
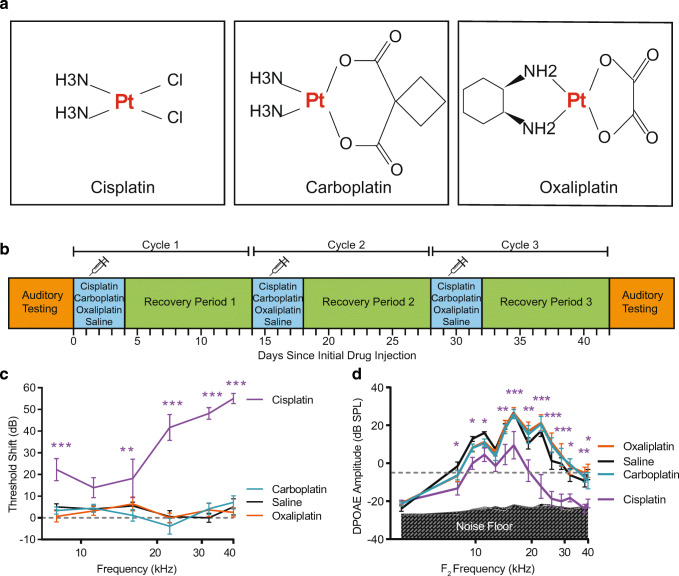
**Cisplatin reduces auditory sensitivity, oxaliplatin and carboplatin do not.** Three platinum-based chemotherapeutic drugs were administered to mice in a cyclic drug administration paradigm at equimolar concentrations. a Cisplatin, carboplatin, and oxaliplatin all contain a central platinum atom. **b** Schematic representation of the experimental protocol including audiometric testing and cyclic drug administration. This figure is adapted from Fernandez et al. 2019. **c** Threshold shift is reported as the difference in ABR thresholds between pre- and post-drug treatment. Mice that received cisplatin (purple) showed significant threshold shifts at 5 of 6 tested frequencies. Hearing thresholds of carboplatin- (blue) and oxaliplatin-treated (orange) mice were not significantly different from those of saline controls (black). Dashed line at 0 dB represents no change in auditory thresholds relative to baseline. **d** OHC function was measured using DPOAEs. Relative to saline-treated control mice (black), cisplatin (purple) caused significantly reduced DPOAE amplitudes at 11 out of 14 tested *f*_2_ frequencies. Neither carboplatin nor oxaliplatin caused decreased DPOAE amplitudes at any frequency. Gray shaded region indicates the mean biological noise floor measured during recordings. Noise floor measurements were taken and plotted for each treatment condition. Dashed line at – 5 dB SPL represents system noise level below which DPOAEs are considered absent. All data are presented as group means ± SEM. *N* = 8–10 mice for all groups. *P < 0.05, **P < 0.01, ***P < 0.001 between cisplatin and saline. (ABR: 2-way ANOVA with Sidak’s test for multiple comparisons, *F*_(3, 181)_ = 116.1, *P* < 0.001; DPOAE: 2-way ANOVA, *F*_(3, 444)_ = 68.19, *P* < 0.001)

Cisplatin is used to treat cancer in both adult and pediatric patients (reviewed in Langer et al. 2013). Cisplatin has significant side effects including neurotoxicity, nephrotoxicity, myelosuppression, and ototoxicity (O'Dwyer et al. 2000; Rabik and Dolan 2007). Cisplatin-induced ototoxicity occurs in 40–60 % of treated adult patients (van Zeijl et al. 1984; Frisina et al. 2016). In pediatric populations, cisplatin causes hearing loss in 20–70 % of patients with an incidence of 28 % of lesions being moderate to severe (Yancey et al. 2012). The resulting hearing loss is permanent and can continue to progress after the cessation of cisplatin treatment (Bertolini et al. 2004; Peleva et al. 2014). Hearing loss can result in reduced quality of life for cancer survivors (Konrad-Martin et al. 2018), and it can disrupt development of speech and language skills in children (Gurney et al. 2007; Lieu et al. 2010; Connor et al. 2006; Blamey et al. 2001). In contrast, carboplatin and oxaliplatin, two related platinum-containing anti-cancer drugs (Fig. 1a), are less ototoxic than cisplatin (Fetoni et al. 2016; Hellberg et al. 2009). The mechanisms underlying this differential sensitivity are poorly understood.

In humans, cisplatin-induced hearing loss is usually bilateral and more severe at higher frequencies (Schaefer et al. 1985; Schell et al. 1989). Higher cumulative cisplatin doses are correlated with more severe hearing loss (Blakley and Myers 1993; Bokemeyer et al. 1998; Biro et al. 2006; Zuur et al. 2006). Approximately 50 % of pediatric medulloblastoma patients experience a clinically significant hearing loss within 2 years of completing cisplatin chemotherapy (Bass et al. 2014). In animal models cisplatin administration results in death of sensory hair cells, with outer hair cells (OHCs) being more susceptible than inner hair cells (IHCs) (Roy et al. 2013; Fernandez et al. 2019; Fernandez et al. 2020; Laurell et al. 2000; Hellberg et al. 2009; Laurell and Bagger-Sjoback 1991; reviewed in Schacht et al. 2012). Cisplatin also damages spiral ganglion neurons and cells of the stria vascularis (Laurell and Engstrom 1989; Laurell et al. 2007). It has been proposed that cisplatin enters cochlear hair cells through organic cation transporters (Ciarimboli et al. 2010).

Carboplatin is a cisplatin analogue that is used to treat a variety of cancer types, including ovarian cancer (Tewari et al. 2019; du Bois et al. 2003; Ozols et al. 2003), lung cancer (Skarlos et al. 1994; Rossi et al. 2012), and some pediatric tumors, including retinoblastoma, osteosarcoma, and neuroblastoma (Clemens et al. 2016; Qaddoumi et al. 2012; Nitz et al. 2013; Peleva et al. 2014). Carboplatin can result in myelosuppression, which can be a dose-limiting toxicity (O'Dwyer et al. 2000; Wagstaff et al. 1989). Carboplatin is less ototoxic in humans than cisplatin (Dean et al. 2008; Bertolini et al. 2004; Fetoni et al. 2016), but ototoxicity has been reported in patients treated with both cisplatin and carboplatin in the same regimen (Waissbluth et al. 2018a, b; Fetoni et al. 2016). Reports of the incidence of carboplatin-induced hearing loss range from as low as 7 % (Peleva et al. 2014) to as high as 46 % (Nitz et al. 2013) of treated patients. Clinical studies suggest that carboplatin-induced ototoxicity may be associated with prior exposure to other ototoxic drugs (Parsons et al. 1998) and with increasing drug dose (Kennedy et al. 1990; MacDonald et al. 1994).

Oxaliplatin is another platinum-based anti-cancer drug that is used to treat colorectal cancers (Pietrangeli et al. 2006; Bleiberg 1998; Becouarn et al. 2001) and gastric and esophageal cancers (Zhang et al. 2019; Al-Battran, et al. 2008). Peripheral neurotoxicity is a dose-limiting side effect of oxaliplatin (Stefansson and Nygren 2016; Gamelin et al. 2002; Saif and Reardon 2005). Despite some reports of isolated instances of oxaliplatin-induced hearing loss (Guvenc et al. 2016; Oh et al. 2013), oxaliplatin is considered non-ototoxic in humans (Pasetto et al. 2006; Yuce et al. 2014). When oxaliplatin is applied directly to rat organ of Corti cultures, the observed cochlear damage is comparable with that caused by cisplatin (Dammeyer et al. 2014; Dalian et al. 2013), suggesting that oxaliplatin may not cross the blood-labyrinth barrier and may therefore be excluded from the cochlea when administered systemically (Hellberg et al. 2009).

We recently examined cisplatin pharmacokinetics in mouse and human inner ears and showed that platinum readily enters the inner ear and is retained there indefinitely (Breglio et al. 2017). Here we have compared the ototoxicity and cochlear uptake of cisplatin, oxaliplatin, and carboplatin using a cyclic drug administration protocol in mice (Fernandez et al. 2019). This approach provides an opportunity to better understand the mechanisms underlying the differential ototoxicity of these three platinum-containing anti-cancer drugs.

## Methods

### Animals

Thirty-one to forty adult male and female CBA/CaJ mice (Jackson Laboratories, Bar Harbor, ME) were used for each experiment. All animal procedures were approved by the NIDCD/NINDS Animal Care and Use Committee (Protocol #1327). All mice were 12–16 weeks of age at the start of the study, as is required by the cisplatin administration protocol (Fernandez et al. 2019; Fernandez et al. 2020).

Mice were monitored daily by study investigators and veterinary team members. Once a day, investigators recorded body weights and assigned each mouse a body conditioning score (BCS) to reflect overall health, including energy level, muscle tone, body fat content, and coat maintenance (Ullman-Cullere and Foltz 1999). Scores range from 1 to 5, a 3 represents a well-conditioned mouse. Mice assigned a BCS score ≤ 2 (under-conditioned) for 24 h were euthanized. Mice were housed individually following completion of baseline audiometric testing and allowed unrestricted access to food and water.

### Hearing Testing

Mice underwent auditory testing that included distortion product otoacoustic emissions (DPOAEs) and auditory brainstem responses (ABRs) prior to and after drug administration. All mice were anesthetized using ketamine (Pulney, Inc., Portland, ME, USA, 100 mg/kg, intraperitoneal (i.p.)) and xylazine (Akorn, Inc., Lake Forest, IL, USA, 10 mg/kg, i.p.). Additional anesthesia was administered at ^1^/_3_-^1^/_2_ the initial dose as necessary during testing. Auditory tests were conducted using Tucker Davis Technologies (TDT, Alachua, FL, USA) hardware and software (BioSigRZ) in a sound attenuated booth (Acoustic Systems, Austin, TX, USA).

DPOAEs were measured from the left ear of each mouse using an ER-10B+ microphone (Etymotic, Elk Grove Village, IL, USA) connected to two MF-1 transducers (TDT). Two primary tones were presented at 14 frequency pairs spanning *f*_2_ = 4 to 40 kHz (L1 = 65 dB SPL and L2 = 55 dB SPL; *f*_2_/*f*_1_ = 1.2) and the amplitude of the DPOAE at 2*f*_1_ − *f*_2_ was recorded. Mean noise levels were calculated based on levels sampled at six surrounding frequencies. Mean emissions and biological noise floor levels were calculated for each treatment group and plotted relative to each other.

ABR testing was conducted immediately after the DPOAE test was completed in the same ear from which the DPOAEs were measured. Responses were recorded from subdermal needle electrodes (Rhythmlink, Columbia, SC, USA) placed at the vertex (noninverting), under the test ear (inverting), and at the base of the tail (ground). Tone-burst stimuli (Blackman window, 3 ms duration, 1.5 ms rise/fall) were presented at a rate of 29.9/s with alternating polarity via a closed-field TDT MF-1 speaker at 8, 11.2, 16, 22.4, 32, and 40 kHz. Averaged waveforms from 1024 presentations were acquired, amplified (20x), filtered (0.3–3 kHz), and digitized (25 kHz). Stimuli at each frequency were presented at 90 dB and decreased until an ABR waveform was no longer present. Threshold was determined by a visual inspection using the lowest sound intensity that resulted in an identifiable waveform with recognizable components. All threshold determinations were verified by an additional investigator.

When testing was complete, Antisedan (Zoetis, Parsippany, NJ, USA, subcutaneous) was injected to reverse the effects of anesthesia. Antisedan was dosed according to the NIDCD Veterinary and Husbandry Care Program guidelines based on body weight: mice weighing 15–19.9 g received 0.015 ml; mice weighing between 20 and 24.9 g received 0.01 ml; mice weighing 25–29.9 g received 0.025 ml, and mice weighing 30–34.9 g received 0.03 ml. In addition, all mice received 0.3 ml saline subcutaneously at the time of Antisedan injection. Mice were placed in a cage devoid of bedding on a warming pad to fully recover before being returned to their original cage and to the animal facility.

### Cyclic Drug Administration Protocols

Mice underwent three rounds of platinum-containing drug administration separated by 10-day recovery periods (Roy et al. 2013; Breglio et al. 2017; Fernandez et al. 2019; Fernandez et al. 2020) (Fig. 1b). A cycle is defined as four consecutive days of once-daily platinum drug injections followed by 10 days of recovery. This cycle was repeated three times for a total of 42 days.

On each day of the protocol, mice received fluid and nutritional support as previously described in detail by Fernandez et al. (2019). Fluid support consisted of 1 mL of 0.9 % saline injected subcutaneously once per day and 1 mL of Normasol (Hospira, Inc., San Clemente, CA, USA) injected subcutaneously once per day, each separated by 6–8 h. Nutritional support consisted of 0.25 mL STAT high-calorie liquid supplement (PRN Pharmacal, Pensacola, FL, USA) delivered into the mouth twice per day. In addition, food pellets (Zeigler Bros, Garnders, PA, USA), bacon softies (Bio-Serv, Flemington, NJ, USA), and Diet Gel Recovery cups (Clear H_2_0, Portland, ME, USA) were placed on each cage floor.

### Equimolar Cyclic Drug Administration Experiment

Our first experiment in this study compared the ototoxicity profiles of carboplatin and oxaliplatin to cisplatin when the drugs were delivered in equimolar doses. Even though the molecules of each drug are of different molecular weights (cisplatin = 300.01 g/mol, carboplatin = 371.25 g/mol, oxaliplatin = 397.29 g/mol), equimolarity ensures the same amount of platinum is delivered to each animal regardless of its treatment group. Forty 12-week-old CBA/CaJ mice (20 males and 20 females) were divided into four groups based on drug treatment: cisplatin, carboplatin, oxaliplatin, or saline-treated controls.

Cisplatin (1.0 mg/ml; WG Critical Care, LLC, Paramus, NJ, USA) was injected i.p. at a daily dose of 3 mg/kg. Carboplatin (10 mg/mL; Teva Pharmaceuticals, North Wales, PA, USA) was diluted 1:9 in 0.9 % saline yielding a 10 % solution that was injected i.p. at a daily dose of 3.71 mg/kg. Oxaliplatin (5 mg/mL, Teva Pharmaceuticals, North Wales, PA, USA) was diluted 1:4 in 0.9 % saline yielding a 20 % solution that was injected i.p. at a daily dose of 3.97 mg/kg. Cisplatin, carboplatin, and oxaliplatin each contain only one Pt atom per molecule, so the doses administered reflect a daily exposure of 1 ± .01 × 10^−5^ mol Pt/kg mouse. Saline-treated control mice received daily injections of 0.9 % saline at a volume of 3 ml/kg which corresponded to the volume delivered to cisplatin-treated mice. At the conclusion of the 42-day protocol, cisplatin–treated mice had received a cumulative dose of 36 mg/kg; carboplatin-treated mice received 44.52 mg/kg and oxaliplatin-treated mice received 47.64 mg/kg.

One oxaliplatin-treated mouse was euthanized on day 32 of the equimolar drug administration protocol due to a BCS score ≤ 2, and one carboplatin-treated mouse died on day 19. None of the cisplatin-treated animals died or met euthanasia criteria during the protocol.

Following the entire cyclic protocol at equimolar concentrations all ABR thresholds were confirmed by two investigators, one of whom is a senior audiology clinician-scientist. When auditory thresholds at either the pre- or post-protocol time point were uninterpretable or inconclusive, that animal’s threshold shift was excluded. Based on these criteria, across all frequencies, 4 % of cisplatin threshold shifts were excluded, 10 % of carboplatin threshold shifts were excluded, 6 % of oxaliplatin threshold shifts were excluded, and 2 % of control threshold shifts were excluded.

### Acute Drug Administration Experiment

To better assess the short-term pharmacokinetics of platinum-based drugs in the inner ear we performed an acute time course experiment of drug injections followed by measurement of platinum levels in inner ear tissues using inductively coupled plasma mass spectrometry (ICP-MS, see below). Thirty-one mice were divided into 8 groups of 3–4 mice each. Each group was assigned to one treatment condition (cisplatin, carboplatin, oxaliplatin, or saline-control) and one time point for measurement (1-h post injection or 24-h post injection). To assess the precise timing of uptake of these three drugs, we injected the equivalent of one cycle of equimolar administration of either cisplatin (12 mg/kg), carboplatin (14.84 mg/kg) or oxaliplatin (15.88 mg/kg) in a single bolus. Saline-treated mice were injected with 12 ml/kg of 0.9 % saline, which corresponded to the volume delivered to the cisplatin-treated mice**.**

Either 1 h or 24 h after the drug injection, mice were euthanized, and ears were dissected and decalcified as described below. For each animal, one ear was analyzed by ICP-MS for whole cochlea samples and the other ear was microdissected into stria vascularis, organ of Corti, and spiral ganglion neuron samples for ICP-MS measurement of platinum levels.

### Clinically Proportional Cyclic Drug Administration Experiment

Our next experiment compared the ototoxicity profiles of carboplatin and oxaliplatin to cisplatin when the drugs were delivered in cumulative doses that reflect the relative proportions at which these drugs are administered clinically. Here, we again used the 3-cycle drug administration protocol described above. Thirty-six (18 male and 18 female) CBA/CaJ mice were divided into four groups based on drug treatment: cisplatin, carboplatin, oxaliplatin, or saline-treated controls.

The cisplatin dosing strategy of 3 mg/kg/day (36 mg/kg cumulative) has been optimized to result in hearing loss that is similar to clinical cisplatin ototoxicity with low risk for animal mortality (Fernandez et al. 2019; Fernandez et al. 2020). Carboplatin is used clinically at doses that are approximately 5–6 times higher than those used for cisplatin (Ozols et al. 2003; du Bois et al. 2003; Fujiwara 2008). In this experiment, carboplatin (10 mg/mL; Teva Pharmaceuticals, North Wales, PA, USA) was diluted 1:1 in 0.9 % saline yielding a 50 % solution that was administered at a daily dose of 15 mg/kg for a cumulative dose of 180 mg/kg. In this experiment, the ratio of the cumulative dose of carboplatin to cisplatin was 5:1.

In the clinic, oxaliplatin is administered to patients at doses that are approximately 0.5–2.57 times higher than those used for cisplatin (Bokemeyer et al. 1998; Uchida et al. 2014; Al-Battran et al. 2008). Here, oxaliplatin (5 mg/mL, Teva Pharmaceuticals, North Wales, PA, USA) was diluted 1:1.77 in 0.9 % saline yielding a 36.1 % solution that was administered at a daily dose of 5.4 mg/kg for a target cumulative dose of 64.8 mg/kg. The ratio of the target cumulative dose of oxaliplatin to cisplatin was 1.8:1. One animal (11 %) died during the first recovery period having received a cumulative dose of 21.6 mg/kg oxaliplatin, and the remaining 8 animals (89 %) were euthanized after receiving BCS scores of ≤ 2 during the second recovery period having received a cumulative dose of 43.2 mg/kg of oxaliplatin. Necropsy reports on these animals indicated thrombocytopenia, a side effect of oxaliplatin that is also observed in humans (Honda et al. 2019; Suh et al. 2014; Woo et al. 2015).

During the clinically proportional dosing experiment, saline-treated control mice again received injections of 0.9 % saline at a volume of 3 ml/kg, corresponding to the volume delivered to the cisplatin-treated group of mice**.**

### Histology

Within 14 days of auditory testing at the end of both cyclic drug administration protocols, mice were euthanized via carbon dioxide asphyxiation followed by decapitation. Cochleas were rapidly dissected and perfused with 4 % paraformaldehyde (PFA) at 4 °C through the round and oval windows and then post-fixed for 1 h at room temperature or overnight at 4 °C. Fixed tissue was decalcified in 0.5 M EDTA for 48 h at room temperature or for up to 96 h at 4 °C.

Fixed ears were assigned to one of three possible preparations for further analysis: (1) immunostaining of cochlear whole mounts, (2) ICP-MS measurement of platinum levels in whole cochlea samples, or (3) ICP-MS measurement of platinum levels in microdissected stria vascularis, organ of Corti, and spiral ganglion neuron samples. During the clinically proportional cyclic drug administration experiment, no tissue was sent for ICP-MS on whole cochleas to preserve the number of samples available for microdissection. No two ears from the same animal were directed to the same type of sample preparation.

The cochleas of ears processed for whole mounts were microdissected in 1× phosphate-buffered saline (PBS) into 5 isolated turns to be immunostained and imaged (Massachusetts Eye and Ear 2020a). Tissue was incubated in blocking solution (5 % normal horse serum in 1X PBS (NHS, Sigma-Aldrich, St. Louis, MO, USA) and Triton X-100 (Sigma-Aldrich; 1:300)) for 1 h and then rinsed for 15 min in PBS. Cochlear turns were immunostained with antibodies to (1) myosin-VIIa (rabbit anti-myosin-VIIA; Proteus Biosciences, Ramona, CA; 1:200) and (2) C-terminal binding protein 2 (mouse anti- CtBP2; BD Biosciences, San Jose, CA; used at 1:200) with secondary antibodies coupled to Alexa Fluors 647 (Invitrogen; used at 1:200) and 568 (Invitrogen; used at 1:1000) respectively. In addition, cochlear turns were stained for actin using Alexa Fluor 488-conjugated phalloidin (Invitrogen; used at 1:50) for the equimolar cyclic drug administration experiment. Due to changes in microscope settings, this antibody was used at 1:1000 for the clinically proportional cyclic drug administration experiment. All antibodies were diluted in 1 % NHS and 30 % Triton X-100. Immunostained tissue was mounted on glass slides using Fluoromount-G (Southern Biotech, Birmingham, AL, USA).

A cochlear frequency map was created via a custom plug-in for ImageJ (Massachusetts Eye and Ear 2020b). Confocal z-stacks (step size of 0.2 μm) corresponding to representative apical (8 kHz), middle (16 kHz), and basal (44 kHz) regions from each ear were collected using an LSM 780 laser scanning confocal microscope (Carl Zeiss AG, Oberkochen, Germany) in a 1024 × 1024 pixel raster (135 μm^2^) using an oil-immersion objective (× 63) of numerical aperture 1.4. IHCs and OHCs were counted in 75-μm-long stretches of the basilar membrane based on the nuclei labeled with C-terminus binding protein 2 (CtBP2).

### Platinum Measurements

The platinum content in our cochlear samples was normalized to sulfur content which is abundant in biological tissue samples. The cochleas of ears processed for ICP-MS were subdivided into two sets—whole cochleas and tissue that was microdissected into organ of Corti, stria vascularis, and spiral ganglion neuron (SGN) samples. All samples were submerged in 100 μl ultra-pure H_2_O (Invitrogen, Carlsbad, CA, USA) during preparation and then dehydrated at 60 °C using a Vacufuge (Eppendorf, Hauppauge, NY, USA) before freezing at − 80 °C until all tissues were ready for ICP-MS analysis.

Samples for analysis by ICP-MS were labeled by the tissue type (whole cochlea, stria vascularis, organ of Corti, or spiral ganglion neurons) and by the animal ID number. The investigators performing the ICP-MS analyses were blinded to the treatment group (cisplatin, carboplatin, oxaliplatin, or saline-treated controls). A NexION 350D inductively coupled plasma mass spectrometer (Perkin Elmer) was used for all platinum measurements. Samples were digested in 50 μl of trace metal nitric acid (Fisher Chemical) and incubated for 20 min at 65 °C. An equal volume of hydrogen peroxide (Optima Grade, Fisher Chemical) was added and the incubation was repeated. Samples were then diluted 1:20 in water. Quantitative analyses were performed in dynamic reaction mode (DRC) with oxygen as the reaction gas at a flow rate of 1.2 ml/min. Single element standards (Perkin Elmer) for platinum (0.5 ppt to 10 ppb) and sulfur (5 ppb to 2 ppm) were used to generate standard curves. This quantitative method was used to measure platinum (Pt 195) and sulfur (as S 48). Platinum values were normalized to sulfur concentration for each sample.

### Statistical Methods

All data presented are group means ± SEM. Statistical tests were conducted using GraphPad Prism 7 software. Significance was set at alpha = 0.05. Two-way ANOVA followed by either Sidak’s or Dunnett’s test for multiple comparisons was used to determine statistical significance for all experiments.

We compared changes in auditory sensitivity between males and females in all drug treatment groups at all concentrations using an unpaired, two-tailed, Student’s *t* test with significance set at alpha = 0.05. After identifying that there was no statistically significant difference between males and females in any dataset, we combined data from animal subjects of both sexes in all analyses.

## Results

### At Equimolar Concentrations, Cisplatin Causes Reduced Auditory Sensitivity, While Oxaliplatin and Carboplatin Do Not

Following the 42-day cyclic drug administration protocol at equimolar concentrations, cisplatin-treated mice showed elevated thresholds across frequencies (2-way ANOVA, *F*_(3,181)_ = 116.1, *P* < 0.001) (Fig. 1c). Post-hoc testing showed that at five of the six tested frequencies (8, 16, 22.4, 32, and 40 kHz), threshold shifts for cisplatin-treated mice were significantly greater relative to those of saline-treated control mice. Among the frequencies where threshold shifts were significant, mean shifts ranged from 18 to 55 dB with an average threshold shift of 37.03 ± 16.15 dB across frequencies. Larger threshold shifts were observed in the higher frequencies. In contrast to cisplatin, neither carboplatin- nor oxaliplatin-treated mice displayed threshold shifts that differed significantly from saline-treated control mice at any frequency (Fig. 1c and Table 1). These results indicate that at our given cisplatin dose of 3 mg/kg/day, cisplatin causes significant hearing loss, and carboplatin and oxaliplatin do not cause hearing loss when administered systemically at equimolar concentrations as cisplatin.

**Table 1:** Only cisplatin causes changes in auditory sensitivity as measured by ABR. *P* values were generated by a 2-way ANOVA with Sidak’s test for multiple comparisons for determining statistical significance between threshold measurements in drug-treated mice relative to saline-treated controls in both the equimolar (Fig. 1c) and clinically proportional (Fig. 5a) cyclic drug administration protocols

Frequency	ABR mean ± SD threshold shift (dB) relative to baseline (*P* value relative to saline-treated controls)					
	8 kHz	11.2 kHz	16 kHz	22.4 kHz	32 kHz	40 kHz
Cisplatin equimolar	22.2 ± 15 *P* = 0.0008	13.9 ± 13.1 *P* = 0.0980	18.1 ± 23.6 *P* = 0.0264	41.7 ± 17.9 *P* < 0.0001	48.1 ± 8.9 *P* < 0.0001	55 ± 8.2 *P* < 0.0001
Cisplatin clin.prop	28.6 ± 9.5 *P* < 0.0001	23.6 ± 20.5 *P* < 0.0001	28.6 ± 20.9 *P* < 0.0001	53.6 ± 14 *P* < 0.0001	45.8 ± 3.8 *P* < 0.0001	49.2 ± 6 *P* < 0.0001
Carboplatin equimolar	3.3 ± 5 *P* = 0.9778	4.4 ± 5.2 *P* = 0.9996	1.1 ± 7.8 *P* = 0.7174	− 3.9 ± 10.8 *P* = 0.7174	4.2 ± 6.5 *P* = 0.8086	7.1 ± 8 *P* = 0.9651
Carboplatin clin. prop	3.1 ± 4.6 *P* = 0.9476	1.9 ± 5.3 *P* = 0.8311	−0.6 ± 5.7*P* = 0.8758	5 ± 7.6 *P* = 0.2543	1.3 ± 5.2 *P* = 0.8961	1.9 ± 6.5 *P* = 0.9149
Oxaliplatin equimolar	0.6 ± 7.3 *P* = 0.7386	3.1 ± 6 *P* = 0.9969	6.4 ± 8.5 *P* = 0.9967	0 ± 5.8 *P* = 0.9995	3.6 ± 8 *P* = 0.8351	2.5 ± 6 *P* = 0.9406
Oxaliplatin clin. prop	Could not test due to systemic oxalipatin toxicity					

Distortion product otoacoustic emissions (DPOAE) were assessed as an indirect measure of OHC function. Figure 1 d shows DPOAE amplitudes as a function of *f*_2_ frequency at the end of the cyclic drug administration protocol. Cisplatin-treated mice had significantly reduced DPOAE amplitudes at 12 of the 14 frequencies tested (2-way ANOVA, *F*_(3, 444)_ = 68.19, *P* < 0.001). At frequencies where the DPOAE amplitude reduction was significant, mean reductions calculated as the negative difference relative to saline-treated controls ranged from 11.54–29.32 dB SPL, and the average decrease was 16.13 ± 5.04 dB across frequencies. The greatest reductions in DPOAE amplitudes were observed in the higher frequencies The DPOAEs measured from carboplatin- and oxaliplatin- treated mice did not vary significantly from saline-treated controls at any frequency. These data indicate that cisplatin resulted in OHC dysfunction and/or loss, while carboplatin and oxaliplatin did not.

### Only Cisplatin Causes Cochlear Outer Hair Cell Death

**Fig. 2:**
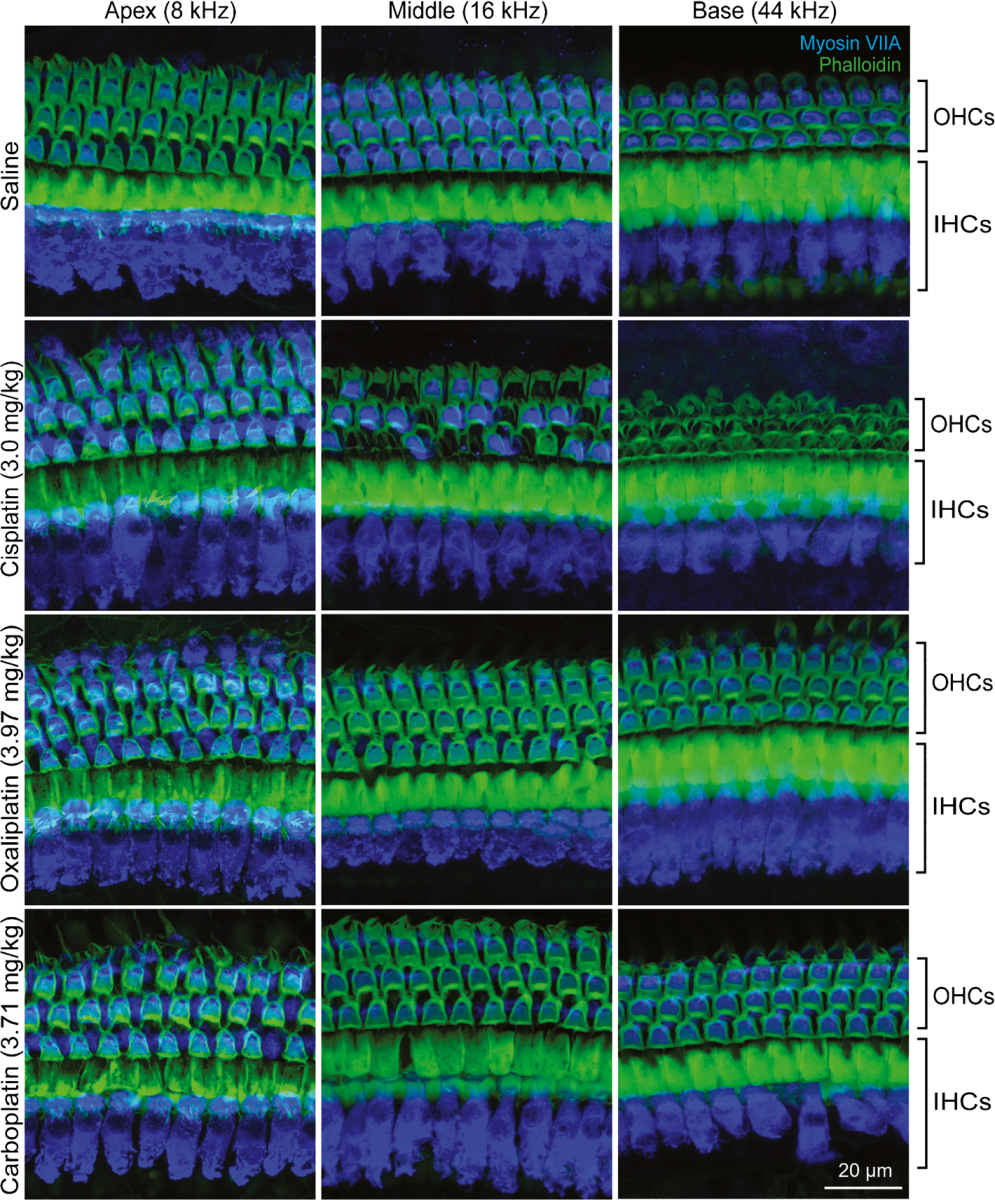
**Cisplatin causes death of outer hair cells (OHCs); oxaliplatin and carboplatin do not.** Isolated cochlear turns were stained for myosin-VIIA (blue) and phalloidin (green). Cochlear inner and outer hair cells were imaged at three distinct locations along the basilar membrane. Saline-treated control mice showed no loss of inner or outer hair cells. In cisplatin-treated mice, inner hair cells (IHCs) remained intact while outer hair cells were missing in the middle and basal cochlear turns. Oxaliplatin and carboplatin did not result in inner or outer hair cell loss in any cochlear turn. Scale bar = 20 μm and applies to all panels

**Fig. 3:**
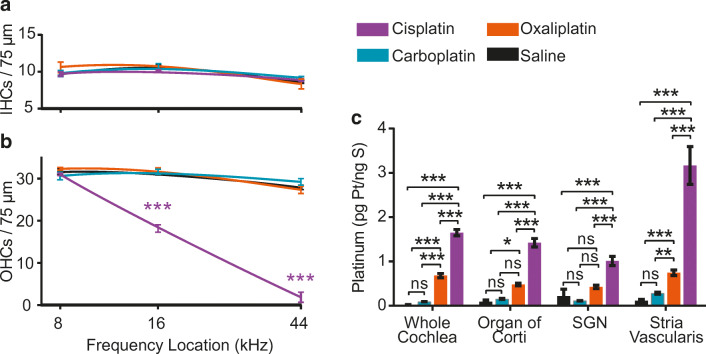
**Cisplatin kills outer hair cells and results in the highest levels of cochlear platinum. **Cisplatin was administered at a daily dose of 3 mg/kg, carboplatin was administered at a daily dose of 3.71 mg/kg and oxaliplatin was administered at a daily dose of 3.97 mg/kg. These equimolar doses were chosen so each animal was exposed to the same amount of platinum regardless of the treatment group to which it was assigned. **a** Inner hair cell counts from mice in all drug administration groups. No significant differences in IHC density were found between saline controls and drug-treated groups at any cochlear location. **b** Outer hair cell counts from mice in all drug administration groups. At middle and basal cochlear turns, there was significant OHC loss in cisplatin-treated mice compared with saline-treated controls. Cisplatin caused 39 % loss of OHCs in the middle turn and 93 % loss of OHCs in the basal turn of the cochlea. Oxaliplatin and carboplatin treatment did not result in loss of outer hair cells. Data displayed are group means ± SEM. *N* = 3–7 cochleas per drug condition. 2-way ANOVA with Sidak’s test for multiple comparisons (*F*_*(3, 51)*_ = 263.3, *P* < 0.001).**c** After three cycles of drug administration, whole cochleas and microdissected cochlear samples were analyzed for platinum content using ICP-MS. Cochleas from cisplatin-treated animals contained significantly increased platinum levels relative to controls in all tissues, including whole cochleas and microdissected organ of Corti, SGN, and stria vascularis, with highest levels in stria vascularis. Tissues from oxaliplatin-treated mice contained significantly increased levels of platinum relative to controls in whole cochlea, organ of Corti, and stria vascularis. In cochleas from carboplatin-treated mice, no significant elevation in platinum was detected in any samples relative to controls. There was a significant difference in platinum accumulation across tissue types (2-way ANOVA with Sidak’s test for multiple comparisons, *F*_*(3, 88)*_ = 32.07, *P* < 0.001) and drug types (2-way ANOVA with Sidak’s test for multiple comparisons, *F*_*(3, 88)*_ = 215.6, *P* < 0.001). Data displayed are group means ± SEM. *N* = 5–9 samples per condition and tissue type. *P < 0.01, ***P < 0.001

Hair cell counts were generated from locations corresponding to representative apical (8 kHz), middle (16 kHz), and basal (44 kHz) turns of the cochlea for IHCs and OHCs (Fig. 2). Frequency maps were generated using an ImageJ plug-in (Massachusetts Eye and Ear 2020b). The number of IHCs did not differ from saline controls among any of the platinum drug treatment groups (2-way ANOVA, *F*_*(3, 51)*_ > = 1.082, *P* = 0.3652,) (Fig. 3a). There were significant differences between cisplatin-treated mice and saline controls in the number of OHCs present in the middle and basal regions of the cochlea (2-way ANOVA, *F*_*(3, 51)*_  = 263.3, *P* < 0.001). There was a 39 % loss of OHCs in the middle turn and a 93 % loss of OHCs in the basal turn following cisplatin exposure. The number OHCs present did not differ significantly between carboplatin- or oxaliplatin-treated mice and saline controls at any location (Fig. 3b). These histological findings agree with the functional data and with our previous studies on cisplatin (Fernandez et al. 2019; Fernandez et al. 2020). Together, these data indicate that when administered at equimolar doses, cisplatin resulted in both OHC death and hearing loss, while carboplatin and oxaliplatin were not ototoxic.

### Platinum Levels in the Inner Ear Are Highest in Cisplatin-Treated Mice

Cochlear uptake of the platinum-containing chemotherapy drugs was measured using ICP-MS to detect the platinum atom at the core of each drug. ICP-MS is a sensitive method for measuring metals that can detect platinum at parts per quadrillion (Brouwers et al. 2008). Platinum measurements are reported as a ratio of picograms (pg) of platinum per nanogram (ng) of sulfur. This ratio was selected because platinum is normally absent in biological tissues whereas sulfur is abundant. The platinum detected in each sample was introduced by our experimental manipulations. Data presented are mean ratios of platinum to sulfur. Cochlear platinum levels were significantly dependent on the type of drug administered (2-way ANOVA, *F*_*(3, 88)*_ = 215.6, *P* < 0.001). Since platinum is not present in normal tissues, platinum levels in saline-treated controls did not rise above minimal noise levels (Tothill et al. 1992). Cisplatin-treated mice had significantly increased platinum levels in whole cochleas relative to control mice (Fig. 3c). Whole cochleas from mice treated with oxaliplatin showed significant platinum levels relative to control but significantly less platinum than cochleas from cisplatin-treated mice. Whole cochleas from carboplatin-treated mice did not contain platinum at levels that were different from controls. Whole cochleas treated with cisplatin had 17 times as much platinum as those treated with carboplatin and 2.5 times as much platinum as those treated with oxaliplatin. Together, these data suggest that at equimolar concentrations, cisplatin readily enters the cochlea, while oxaliplatin enters the cochlea less readily, and carboplatin either does not enter the cochlea or is rapidly cleared after entry.

In order to further localize platinum-containing drugs within the inner ear, some cochleas were microdissected and analyzed for platinum content in the organ of Corti, stria vascularis, and spiral ganglion neurons (SGN) separately. Cochleas of cisplatin-treated mice showed significant platinum levels relative to controls in all three cochlear regions, with highest levels in the stria vascularis (Sidak’s test for multiple comparisons, *P* < 0.001) (Fig. 3c), consistent with our previous report (Breglio et al. 2017). Cochleas from oxaliplatin-treated mice showed significantly elevated platinum levels in organ of Corti and stria vascularis (Sidak’s test for multiple comparisons *P <* 0.05), where platinum content was statistically higher than controls but remained significantly lower than cisplatin-treated mice (Sidak’s test for multiple comparisons, *P* < 0.001). There was no significant increase in platinum in any carboplatin-treated samples relative to controls. Among the three microdissected organs of the cochlea, platinum levels following cisplatin were on average 9.8 ± 4.1 times greater than those following carboplatin and 3.0 ± 0.8 times greater than following oxaliplatin exposure.

### The Differences in Cochlear Platinum Levels Among the Three Platinum-Containing Drugs Are Not Accounted for by Differences in Drug Clearance from the Tissue

**Fig. 4:**
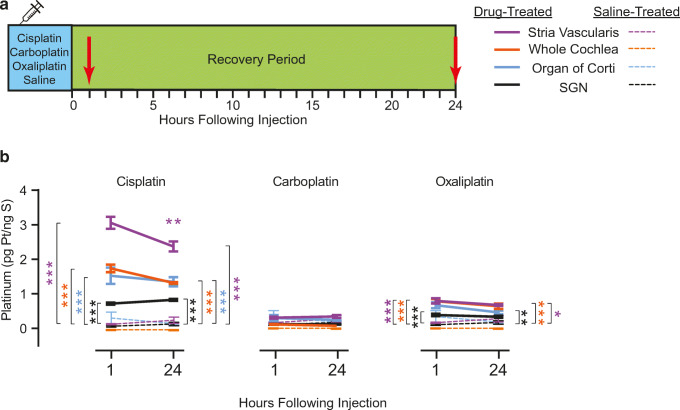
**Drug clearance from the cochlea does not account for the differences in platinum levels. a** In order to determine if the observed differences in cochlear platinum levels were due to differences in drug clearance, we measured cochlear platinum levels 1 h and 24 h after administering the equivalent of one drug injection period (4 days) of equimolar solutions via a single i.p. injection. **b** Platinum levels were measured in four tissue types treated with either cisplatin, carboplatin, or oxaliplatin and compared relative to saline-treated controls. Platinum levels were significantly dependent on drug type at both 1-h (2-way ANOVA, *F*_*(3, 44)*_ = 233.5, *P* < 0.001) and 24-h following injection (2-way ANOVA, *F*_*(3, 44)*_ = 50.66, *P* < 0.001). Dunnett’s post-hoc test for multiple comparisons revealed significant platinum uptake in all 4 cisplatin-treated tissue types, no carboplatin-treated tissue types, and some oxaliplatin-treated tissue types (spiral ganglion neurons, stria vascularis, and whole cochleas). Solid lines represent platinum levels detected in drug-treated samples; dashed lines represent platinum levels detected in saline-treated control samples. Data displayed are group means ± SEM. *N* = 3–4 samples per treatment condition and time point. *P < 0.05, **P < 0.01, ***P < 0.001

Our previous data indicate that platinum levels in the cochlea are elevated 1 h after cisplatin administration, and they remain elevated indefinitely (Breglio et al. 2017). Our finding that cochleas from mice treated with oxaliplatin or carboplatin contained significantly lower levels of platinum compared with those from mice treated with cisplatin at the end of the 42-day administration period suggests that either (1) oxaliplatin and carboplatin do not enter the cochlea as readily as cisplatin, or (2) oxaliplatin and carboplatin enter the cochlea and then are cleared from the tissue more rapidly than cisplatin. To distinguish between these two possibilities, we administered each platinum-containing drug and measured cochlear platinum levels at short time intervals (1 and 24 h) after injection (Fig. 4a).

We exposed mice to the equivalent of one drug injection period (4 days) of equimolar solutions of each platinum-containing drug via a single i.p. injection. Cisplatin was delivered at 12 mg/kg, carboplatin was delivered at 14.84 mg/kg, and oxaliplatin was delivered at 15.88 mg/kg. One ear from each mouse was prepared for whole cochlea ICP-MS, and the other ear was microdissected into organ of Corti, stria vascularis, and spiral ganglion neurons for ICP-MS. Acute platinum uptake was assessed at 1 h and 24 h following a single injection of each drug for each tissue type relative to saline-treated controls. Platinum levels varied significantly depending on the specific platinum drug at both 1 h (2-Way ANOVA, *F*_*(3,44)*_ = 233.5, *P* < 0.001) and at 24 h (2-Way ANOVA, *F*_*(3,44)*_ = 50.66, *P* < 0.001). At both timepoints, platinum levels were significantly elevated relative to saline in all four tissue types in mice treated with cisplatin and in SGN, stria vascularis, and whole cochlea of oxaliplatin-treated mice (Dunnett’s test multiple comparisons, *P* < 0.01). No significant elevation in platinum levels was detected for carboplatin-treated mice in any tissue sample at either 1 h or 24 h. While platinum levels in cisplatin-treated stria vascularis remained significantly higher than saline-treated controls at both time points, there was a statistically significant decrease in platinum between 1 and 24 h in stria vascularis samples (2-Way ANOVA, *F*_*(1,12)*_ = 7.558, *P* = 0.0117). This fluctuation in strial platinum levels following a single injection over 24 h was similarly observed by Breglio et al. (2017). There were no other statistically significant differences in platinum levels detected between 1 and 24 h time points within a specific tissue type. Thus, cisplatin readily enters the cochlea by 1 h after injection, and platinum levels remain elevated 24 h later. Carboplatin does not enter the cochlea, while oxaliplatin enters the cochlea and is not cleared by 24 h. Taken together these data indicate that drug clearance does not account for the differential levels of platinum we observed at the end of the 42-day cyclic drug administration protocol. Our data are consistent with a model in which the differential ototoxicity of these three drugs is not attributable to differential clearance of the drugs but instead is due to differential uptake of the three drugs by the inner ear.

### At Clinically Proportional Concentrations, Carboplatin Does Not Cause Hearing Loss

The above experiments used cisplatin, carboplatin, and oxaliplatin in equimolar concentrations. In the clinic, these three drugs are not often used to treat the same types of cancer. When they are, the drugs are not delivered to patients in equimolar concentrations; rather, carboplatin and oxaliplatin are administered at doses several times greater than that of cisplatin. In these cases, a ratio of the relative doses between cisplatin and either carboplatin or oxaliplatin can be determined. In clinical studies in which the efficacy of carboplatin was directly compared with cisplatin, carboplatin was administered at doses 5–6 times higher than cisplatin (Ozols et al. 2003; Du Bois et al. 2003). Similarly, oxaliplatin is administered at doses 1.7–2.5 times higher than cisplatin (Yu et al. 2015; Li et al. 2011a; Weissman et al. 2011; Al-Battran et al. 2008). We kept the cisplatin dose unchanged from the equimolar experiments because this regimen has been optimized to result in little to no morality while yielding a reliable hearing loss in mice (Breglio et al. 2017; Fernandez et al. 2019; Fernandez et al. 2020). We selected carboplatin and oxaliplatin doses that reflect the proportions of these drugs administered clinically. Cisplatin was administered at 3 mg/kg/day yielding a target cumulative dose of 36 mg/kg over the course of the full three-cycle administration protocol. Carboplatin was administered at 15 mg/kg/day (5 times higher than cisplatin), yielding a target cumulative dose of 180 mg/kg over the course of the full cyclic administration protocol. Finally, oxaliplatin was administered at 5.4 mg/kg/day (1.8 times higher than cisplatin), yielding a target cumulative dose of 64.8 mg/kg over the course of the full cyclic administration protocol.

**Fig. 5:**
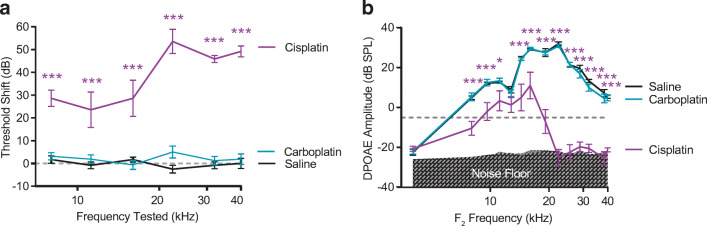
At clinically proportional concentrations, carboplatin does not cause auditory dysfunction. **a** Following the clinically proportional drug administration protocol, ABR threshold shifts were measured for animals treated with cisplatin (purple), carboplatin (blue) delivered at a dose 5 times greater than cisplatin, or saline controls (black). Cisplatin-treated animals showed increased threshold shifts relative to saline-treated controls at all tested frequencies (2-way ANOVA with Sidak’s test for multiple comparisons, *F*_(2, 106)_ = 220.1, *P* < 0.001). Carboplatin treatment did not result in threshold shifts at any tested frequency despite the higher dose of carboplatin. **b** DPOAEs were measured as an indirect assessment of OHC function. Cisplatin-treated mice had reduced DPOAE amplitudes at 12 of the 14 tested *f*_2_ frequencies, and carboplatin-treated animals showed no changes relative to saline-treated controls at any frequency (2-Way ANOVA with Sidak’s test for multiple comparisons, *F*_(2,252)_ = 382.8, *P* < 0.0001). Data displayed are group means ± SEM. *N* = 6–8 subjects per treatment condition. *P < 0.05, **P < 0.01, *** *P* < 0.001

Under these “clinically proportional” conditions, only cisplatin-treated mice had significant threshold shifts from baseline (2-Way ANOVA, *F*_(2, 106)_ = 220.1, *P* < 0.001) (Fig. 5a). At all six of the tested frequencies, mice treated with cisplatin showed significant mean threshold shifts ranging from 23.57–49.17 dB with an average threshold shift across frequencies of 38.21 ± 12.76 dB relative to saline-treated control mice. Even when the cumulative dose of carboplatin was 5× greater than that of cisplatin (180 mg/kg versus 36 mg/kg), there were no significant threshold shifts at any tested frequency relative to saline-treated controls (Fig. 5a and Table 1). A similar pattern of damage was seen in the measurements of OHC function via DPOAE amplitudes collected at the conclusion of the clinically proportional administration protocol (2-way ANOVA, *F*_(2,252)_ = 382.8, *P* < 0.001) (Fig. 5b). The DPOAE amplitudes for cisplatin-treated mice differed significantly from those of saline-treated controls at 12 of the 14 frequencies tested. At frequencies where the DPOAE amplitude reduction was significant, mean reductions calculated as the negative difference relative to saline-treated controls ranged from 9.27–57.29 dB with an average decrease of 28.73 ± 14.14 dB across frequencies. There were no significant differences in DPOAE amplitudes following carboplatin administration even at the increased drug dosage. Taken together these data indicate that when carboplatin is administered at five times the dose of cisplatin (as is common clinically), no changes in hearing sensitivity or outer hair cell function are observed in carboplatin-treated mice.

We were unable to obtain physiological measurements for oxaliplatin-treated mice treated at a daily dose of 5.4 mg/kg because one animal died after the first cycle of drug administration and the remaining eight animals met the euthanasia criterion of a BCS ≤ 2 by the second recover period. Necropsies performed on 2 animals revealed thrombocytopenia. These data indicate that mice cannot be treated with oxaliplatin at daily doses higher than 3.97 mg/kg.

### At Clinically Proportional Concentrations, Only Cisplatin Causes Hair Cell Loss

**Fig. 6:**
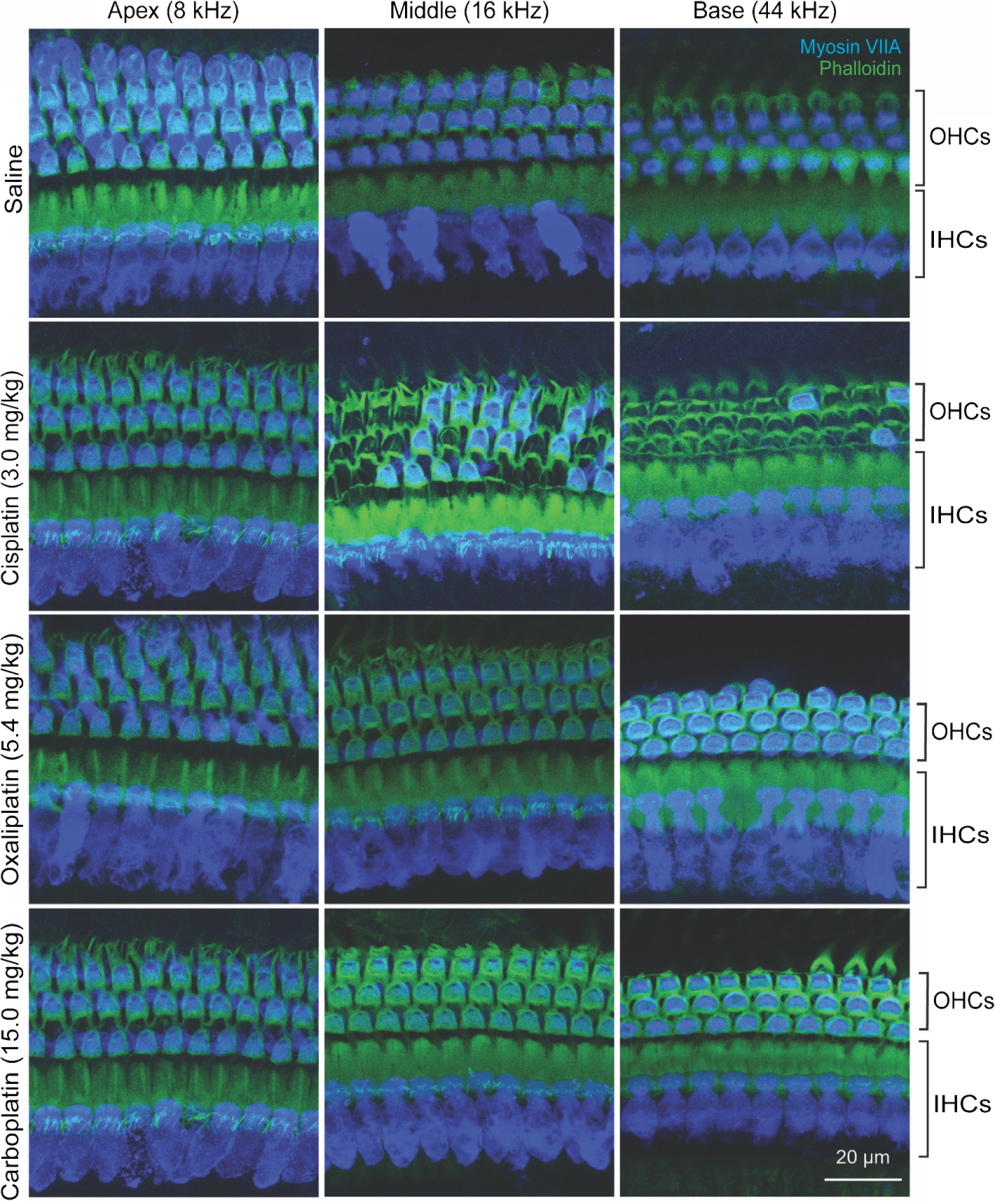
**At clinically proportional doses, only cisplatin causes OHC loss. **Isolated cochlear turns were stained for myosin-VIIA (blue) and phalloidin (green). Cochlear inner and outer hair cells were imaged and counted at three distinct locations along the basilar membrane. Control (saline-treated) mice showed no loss of IHCs or OHCs. Cisplatin-treated mice showed no loss of IHCs and significant loss of OHCs in the basal and middle cochlear turns. Oxaliplatin and carboplatin did not result in IHC or OHC loss in any cochlear turn. Scale bar = 20 μm and applies to all panels

**Fig. 7:**
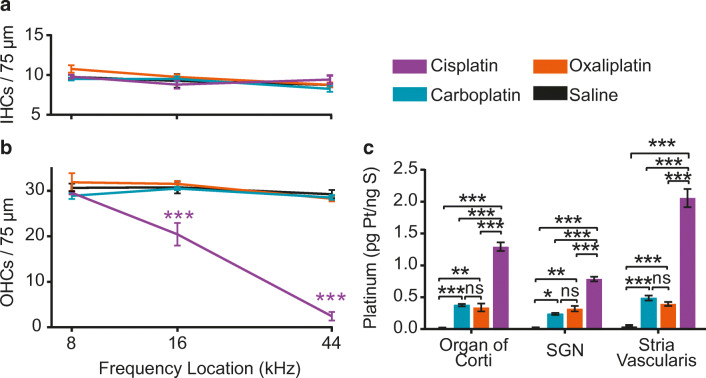
**At clinically proportional doses, neither carboplatin nor oxaliplatin caused a decrease in HC density, and cochlear platinum levels following carboplatin or oxaliplatin treatment remain lower than those of cisplatin. **The cumulative dose of cisplatin remained unchanged relative to the equimolar experiment at 36 mg/kg. Carboplatin was administered at 5 times that dose at 180 mg/kg. Oxaliplatin was administered at 1.2 times the cisplatin dose at 43.2 mg/kg. **a** No significant differences in IHC density were found between saline-treated controls and drug-treated groups at any cochlear location. **b** OHC density was significantly dependent on drug treatment (2-way ANOVA, *F*_*(3, 76)*_ = 73.3, *P* < 0.001). Cisplatin-treated mice showed significant OHC loss at middle (34 %) and basal (92 %) cochlear turns compared with saline controls (Sidak’s test for multiple comparisons, *P <* 0.001). Oxaliplatin or carboplatin treatment did not result in significant loss of OHCs at any cochlear location. Data displayed are group means ± SEM. *N* = 4–8 cochleas per condition. ***P < 0.001. **c** Microdissected cochlear samples were assessed for platinum content using ICP-MS. Cochlear platinum levels were significantly elevated for all samples treated with platinum-containing drugs relative to saline controls (2-way ANOVA with Sidak’s test for multiple comparisons, *F*_*(3, 72)*_ = 358.2, *P* < 0.001). Cochlear platinum levels following carboplatin and oxaliplatin were significantly lower than those following cisplatin. Data presented are mean ± SEM**.** *N* = 5–10 samples per condition. *P < 0.05, **P < 0.01, ***P < 0.001

Hair cell counts were generated from locations corresponding to representative apical (8 kHz) middle (16 kHz) and basal (44 kHz) regions of cochleas from mice treated with clinically proportional concentrations of platinum-containing drugs (Fig. 6). The number of IHCs again did not differ from saline-treated controls in any drug-treated group at any cochlear location (Fig. 7a). OHC density was significantly dependent on drug treatment (2-Way ANOVA, *F*_*(3, 76)*_ = 73.3, *P* < 0.001). There were significant differences between cisplatin- and saline-treated mice in the number of OHCs present in the middle and basal turns of the cochlea (Sidak’s test for multiple comparisons, *P* < 0.001) (Fig. 7b). Cisplatin-treated mice showed 34 % OHC loss in the middle turn of the cochlea and 92 % OHC loss in the basal turn. There were no significant differences in the number of OHCs present in cochleas exposed to carboplatin or oxaliplatin compared with saline controls at any cochlear location. Thus, cisplatin treatment caused significant elevation in auditory thresholds, significant reduction in DPOAE amplitudes, and commensurate OHC loss in the inner ear. By contrast, carboplatin treatment, even at a higher dose, did not cause auditory dysfunction or hair cell death.

Although it was not possible to obtain physiological data for mice exposed to oxaliplatin, we were able to quantify hair cell loss from animals that were exposed to 2 cycles of oxaliplatin administered at 5.4 mg/kg/day of injection for a cumulative exposure of 43.2 mg/kg. This dose represents 1.2x the amount of drug that the cisplatin mice were exposed to in the initial equimolar experiment. Relative to controls, there was no significant decrease in the number of inner or outer HCs present in cochleas from oxaliplatin-treated mice.

### At Clinically Proportional Concentrations, Cochlear Platinum Levels Following Carboplatin and Oxaliplatin Administration Remain Low Compared with Cisplatin

We measured cochlear platinum levels at the end of the clinically proportional drug administration protocol. Overall, cochlear platinum levels were significantly dependent on the type of drug administered (2-way ANOVA, *F*_*(3, 72)*_ = 358.2, *P* < 0.001) (Fig. 7c). Consistent with our previous result, there was statistically significant platinum uptake relative to saline controls in all cochlear regions following cisplatin administration (Sidak’s test for multiple comparisons, *P* < 0.001*)*. Cisplatin caused an average 74.75-fold increase in platinum levels compared with saline-treated controls. When carboplatin was administered with a cumulative dose of 180 mg/kg (5× greater than the cumulative cisplatin dose of 36 mg/kg), there was a significant elevation in cochlear platinum relative to saline controls for all three microdissected organ types of the inner ear (Sidak’s test for multiple comparisons, *P* < 0.05). This contrasts with our experiment in which carboplatin was delivered at a dose that is equimolar to that used for cisplatin (cumulative dose 44.52 mg/kg, see Fig. 3), in which there was no statistically significant elevation in cochlear platinum levels. This suggests that the cochlear uptake of carboplatin is dose dependent. Still, cochlear platinum levels after clinically proportional carboplatin administration are significantly less than after cisplatin administration in organ of Corti, stria vascularis, and SGN (Sidak’s test for multiple comparisons, *P* < 0.001).

Even though oxaliplatin-treated animals did not complete the full 6-week protocol, ears of mice that were euthanized during the second recovery period were harvested and processed for ICP-MS. These animals received a cumulative dose of 43.2 mg/kg oxaliplatin. In cochleas from oxaliplatin-treated mice, we observed significantly increased levels of platinum in the organ of Corti, SGN, and stria vascularis relative to saline-treated controls (Sidak’s test for multiple comparisons, *P* < 0.01), but these levels remained significantly lower than those in cochleas from cisplatin-treated mice (Sidak’s test for multiple comparisons, *P* < 0.001) (Fig. 7c) (average 21.30-fold increase in platinum levels compared with 74.75-fold increase following cisplatin treatment). Even though these animals did not complete the full clinically proportional drug administration protocol, the pattern and location of cochlear platinum uptake following oxaliplatin exposure were consistent with measurements obtained during the equimolar cyclic drug administration protocol where over three cycles, oxaliplatin-treated mice received 47.64 mg/kg. There was no statistically significant difference in platinum accumulation between carboplatin- and oxaliplatin-treated animals in any microdissected organ.

## Discussion

Cisplatin, oxaliplatin, and carboplatin are three platinum-containing anti-cancer drugs with differing ototoxicity profiles. When these drugs were administered in equimolar concentrations, we observed auditory threshold shifts and decreases in DPOAE amplitudes caused by cisplatin, while neither oxaliplatin nor carboplatin caused significant changes in these physiological measures. These data were consistent with commensurate differences in OHC survival, with cisplatin-treated mice showing significant OHC death, while carboplatin- or oxaliplatin-treated mice showed no OHC death. Cisplatin-treated mice showed a significant increase in platinum in all cochlear regions. Oxaliplatin-treated mice showed a significant increase in cochlear platinum in whole cochlea, stria vascularis, and organ of Corti, while carboplatin-treated mice showed no increases in cochlear platinum levels. These data indicate that the differential ototoxicity among these three drugs is likely caused by either differences in cochlear uptake or differences in the rates of clearance of the drugs from cochlear tissues. Data obtained from short-term (1–24 h) drug exposures indicated that cochlear platinum levels detected within 1 h of a drug exposure remain consistent 24 h later. Thus, the differences in cochlear platinum levels among these three drugs are not attributable to differences in the rates of clearance from the cochlea. Our data are consistent with the hypothesis that the differential ototoxicity of these three platinum-containing drugs is caused by differential cochlear uptake of the drugs; cisplatin readily enters the cochlea, while oxaliplatin and carboplatin are largely excluded from the inner ear.

Our first set of experiments used equimolar concentrations of each drug. We chose this paradigm in order to maintain consistency in the amount of platinum each animal was exposed to. However, clinically these drugs are not administered at equimolar doses. Oxaliplatin is typically administered at doses 1.8–2.5 times higher than cisplatin (Bokemeyer et al. 1998; Uchida et al. 2014; Al-Battran et al. 2008), and carboplatin is usually administered at doses 5–6 times higher than cisplatin (Ozols et al. 2003; du Bois et al. 2003; Fujiwara 2008). Therefore, we repeated the cyclic drug administration protocol using cumulative doses that are proportional to those administered clinically in order to determine whether higher doses of oxaliplatin and carboplatin result in drug uptake into the inner ear and/or hearing loss. In cisplatin-treated mice, we again saw a frequency-dependent increase in auditory thresholds as measured by ABR. We also saw a frequency-dependent decrease in DPOAE amplitudes. These results are consistent with those observed in the equimolar dosing experiment and our previous reports. When we administered carboplatin at a cumulative dose of 180 mg/kg, we saw no significant ABR threshold shifts and no decrease in DPOAE amplitudes relative to saline controls at any tested frequency. Moreover, we saw no change in the number of hair cells present in the cochlea and minimal uptake of platinum in the cochlea. Thus, our data indicate that carboplatin does not readily enter the cochlea and results in no observable ototoxicity using these measures. These data are in agreement with several studies showing that at standard doses in adult humans, carboplatin alone does not cause significant hearing loss (Kennedy et al. 1990; Calvert et al. 1982; De Lauretis et al. 1999). It is possible that carboplatin may result in loss of cochlear ribbon synapses or damage to the ascending auditory pathway that might not be evident in our ABR and DPOAE assays. Carboplatin-induced hearing loss has been reported when carboplatin is combined with radiation (Fetoni et al. 2016) and other ototoxic treatments (Waissbluth et al. 2018a, b; Parsons et al. 1998).

In all three experiments, oxaliplatin caused significantly elevated platinum levels relative to saline-treated controls. However, the platinum levels in the inner ears of oxaliplatin-treated mice were never as high as those in cisplatin-treated mice. In both the equimolar and the clinically proportional protocols, samples from oxaliplatin-treated mice had significantly less platinum than those from cisplatin-treated mice (Figs. 3 and 7). These findings suggest that there may be a threshold level of platinum in the inner ear above which ototoxicity occurs and below which damage is undetectable. This idea is supported by several additional lines of evidence. First, previous data from our lab in which ABRs were recorded after each cycle of cisplatin administration (Breglio et al. 2017) indicate that no hearing loss occurs after the first cycle of cisplatin administration, and only minimal threshold shifts occur after the second cycle. Three cycles of cisplatin administration were required before we observed hearing loss that is reminiscent of clinical cisplatin ototoxicity. Second, following IV infusion of oxaliplatin and cisplatin at equimolar concentrations in guinea pigs, Hellberg et al. (2009) observed significantly lower levels of oxaliplatin than cisplatin in perilymph, and no hair cell loss was caused by oxaliplatin. Thus, it appears that there is less ototoxic damage when less platinum enters the cochlea. Third, retrospective clinical studies suggest that incidence of hearing loss increases at higher cumulative doses of cisplatin (Schell et al. 1989; Rademaker-Lakhai et al. 2006). We attempted to determine if increased oxaliplatin doses would reach this threshold and result in elevated ABR thresholds and/or reduced DPOAE amplitudes. Our initial target cumulative dose for oxaliplatin was 64–90 mg/kg. However, none of the mice in this group completed the protocol—they met euthanasia criteria after receiving BCS scores ≤ 2 by the second cycle of oxaliplatin administration. Necropsy results from oxaliplatin-treated mice revealed thrombocytopenia, which is also observed in humans treated with oxaliplatin (Curtis et al. 2006; Jardim et al. 2012). Thus, we were unable to administer oxaliplatin over the 6-week protocol at a daily dose higher than 3.97 mg/kg/day (cumulative dose of 47.64 mg/kg). We previously described in detail the health monitoring and nutritional supplementation procedures required for this cisplatin administration protocol in mice (Fernandez et al. 2019). We adhered to these methods when administering all three drugs in each experiment. By comparison, at the conclusion of the 42-day clinically proportional experiment animals that received cisplatin had average body weights of 78 ± 6 % relative to baseline, and animals receiving carboplatin had average body weights of 102 ± 5 % relative to baseline. All animals in the carboplatin- and cisplatin-treated groups retained body conditioning scores (≥ 3), meeting criteria for continuation in the experiment.

Our studies use drug administration protocols that mimic the clinical delivery of these drugs to patients in that we utilized cycles of drug administration separated by periods of recovery. Earlier studies examining the relationship between platinum drug entry and ototoxicity used daily administration of cisplatin and carboplatin without recovery periods in guinea pigs (Schweitzer et al. 1984; Schweitzer et al. 1986). These studies showed similar patterns of platinum uptake using gamma ray emission analysis, with the inner ear showing more vulnerability to cisplatin ototoxicity than carboplatin, and with platinum levels in stria vascularis being significantly greater than other areas in the inner ear. One study that used ICP-MS to compare platinum uptake in various organs following exposure to cisplatin, carboplatin, and oxaliplatin suggested that oxaliplatin exposure yields the highest levels of platinum in the inner ear (Esteban-Fernandez et al. 2008). This study used single-bolus injections in Wistar rats, and the ratio of oxaliplatin dosage to cisplatin dosage was 5:1. We used the ratio of 1.7:1 which is consistent with clinical studies that directly compared therapeutic efficacy of cisplatin to that of oxaliplatin (Bokemeyer et al. 1998; Uchida et al. 2014; Al-Battran et al. 2008). Notably, Esteban-Fernandez (et al. 2008) administered 28.1 times more carboplatin than cisplatin, and platinum levels from carboplatin-treated samples still did not reach those of samples exposed to cisplatin.

The mechanisms by which platinum-containing drugs enter (or are excluded from) the inner ear are not fully understood. Pharmacologic and genetic studies have implicated organic cation transporters (OCTs) as mediators of cisplatin entry into both the kidney and in the cochlea (Ciarimboli et al. 2010; Ciarimboli 2011; Lanvers-Kaminsky et al. 2015; Hucke and Ciarimboli 2016). Yonezawa et al. (2006) show that cisplatin and oxaliplatin are substrates for human OCTs, while carboplatin is not. Studies using OCT knockout mice in conjunction with a low dose cyclic cisplatin administration protocol suggest functional blocking of OCTs as a strategy to prevent cisplatin ototoxicity (Hucke et al. 2019). It is unclear whether OCTs account for all uptake of platinum in the cochlea, if OCTs are present in all inner ear cell types, or if there are OCT-independent mechanisms of uptake that may contribute to ototoxicity.

A variety of antioxidants have been shown to reduce cisplatin-induced ototoxicity in animal models (Rybak et al. 1999; Lopez-Gonzalez et al. 2000; Choe et al. 2004). Studies that show antioxidants protects against hair cell death in vitro (Kim et al. 2014; Astolfi et al. 2008, Roldán-Fidalgo et al. 2016, reviewed in Sheth et al. 2017) suggest that antioxidant-mediated protection can function downstream of cisplatin entry into the inner ear, since in vitro preparations eliminate the blood-labyrinth barrier and allow cisplatin to directly access hair cells. However, to date no studies have shown that antioxidants reduce cisplatin-induced hearing loss in humans. Given that OCT1/2 knockout mice are resistant to cisplatin-induced hearing loss (Ciarimboli et al. 2010) and that in vitro antioxidants confer protection from cisplatin, the available data are consistent with a model in which antioxidant-mediated protection occurs downstream of cisplatin entering the inner ear.

Our data suggest that stria vascularis samples accumulate higher levels of platinum than other tissue types including the organ of Corti (Figs. 3c, 4c, and 7c). Liu et al. (2016) showed that OHC survival is dependent on normal function of the stria vascularis, and our lab reported reduced endocochlear potentials following cisplatin administration (Breglio et al. 2017). Given that cisplatin treatment caused significant OHC death (Figs. 2, 3b, 6, and 7b) and significant platinum uptake in the stria vascularis, our data do not rule out the possibility that hair cell death is secondary to loss of strial function.

Here we compared auditory dysfunction, hair cell death, and cochlear platinum uptake in mice treated with cisplatin, carboplatin, or oxaliplatin. Our results show ABR threshold elevation, DPOAE amplitude reduction, OHC death, and platinum uptake in cochleas of cisplatin-treated mice. Carboplatin- and oxaliplatin- treated mice did not show changes in hearing sensitivity, and cochlear platinum levels remained lower than those in cisplatin-treated mice. When these drugs are administered in doses proportional to those used clinically, we still see no ototoxicity and relatively little platinum uptake. Overall our data indicate that the differential ototoxicity of these three platinum-containing drugs is due to differences in cochlear uptake.
